# Neuro-motor controlled wearable augmentations: current research and emerging trends

**DOI:** 10.3389/fnbot.2024.1443010

**Published:** 2024-10-31

**Authors:** Haneen Alsuradi, Joseph Hong, Helin Mazi, Mohamad Eid

**Affiliations:** ^1^Engineering Division, New York University Abu Dhabi, Abu Dhabi, United Arab Emirates; ^2^Center for Artificial Intelligence and Robotics, New York University Abu Dhabi, Abu Dhabi, United Arab Emirates

**Keywords:** wearable augmentation, electroencephalography, electromyography, sensory feedback, embodiment

## Abstract

Wearable augmentations (WAs) designed for movement and manipulation, such as exoskeletons and supernumerary robotic limbs, are used to enhance the physical abilities of healthy individuals and substitute or restore lost functionality for impaired individuals. Non-invasive neuro-motor (NM) technologies, including electroencephalography (EEG) and sufrace electromyography (sEMG), promise direct and intuitive communication between the brain and the WA. After presenting a historical perspective, this review proposes a conceptual model for NM-controlled WAs, analyzes key design aspects, such as hardware design, mounting methods, control paradigms, and sensory feedback, that have direct implications on the user experience, and in the long term, on the embodiment of WAs. The literature is surveyed and categorized into three main areas: hand WAs, upper body WAs, and lower body WAs. The review concludes by highlighting the primary findings, challenges, and trends in NM-controlled WAs. This review motivates researchers and practitioners to further explore and evaluate the development of WAs, ensuring a better quality of life.

## 1 Introduction

Throughout history, humans have strived to enhance their physical and cognitive abilities through technological innovations. For example, the invention of vehicles allowed humans to overcome their natural speed limitation, while the emergence of the internet expanded our communication abilities. This practice of enhancing human capabilities through the application of technology falls under the field of *human augmentation* (Guerrero et al., 2022). Within this larger field, human *movement* augmentation (HMA) refers to the enhancement of sensorimotor capabilities, as opposed to cognitive ones, which can include improvement of strength, endurance, and mobility (Cinel et al., 2019). Given the physical nature of this mode of augmentation, implementations within HMA also share this physical characteristic.

HMA can be wearable, designed to provide support or enhancement while attached to the human body. Non-wearable augmentations, on the other hand, enhance human capabilities as standalone devices. Furthermore, the implementation of augmentation can be invasive, requiring medical interventions, or non-invasive, which requires alternative methods to integrate the augmentation with the user. Additionally, the augmentation can be controlled by various agents. An autonomous mode of control operates through an algorithm or model on the device's controller, occasionally accepting user preferences but not as a direct input, primarily relying on its own logic for decisions. A voluntary mode of control involves full user input, often through physiological (such as surface electromyography or electroencephalography) or non-physiological (such as a joystick or pedals) signals.

Given the plethora of implementation taxonomies within HMA, we focus on a specific subset of HMA implementations that have been gaining interest in the community: wearable augmentations (WAs) that are non-invasive and primarily controlled through a voluntary control mode via neurophysiological signals to infer user intent.

### 1.1 Wearable augmentations

WAs refer to a category of HMA devices designed to be physically attached to the human body with the purpose of assisting in performing a variety of physical tasks that would otherwise be impossible for an individual. WAs include both exoskeletons and supernumerary robotic limbs such as fingers, arms, or legs. Exoskeletons are typically designed to provide support to either upper or lower limbs for serving different purposes such as rehabilitation (Cao and Huang, 2020) or augmentation (Kazerooni, 2008). These exoskeletons are designed to fit the natural configuration of the human body, to be worn as external suits, and are equipped with actuators for the purpose of assisting human joints. For example, lower body exoskeletons are designed to aid in weight-bearing, reducing the metabolic cost of locomotion, and facilitating gait rehabilitation (Siviy et al., 2023). On the other hand, upper body exoskeletons are used in enhancing load lifting capabilities, increasing strength and endurance. As such, they often provide support to workers and laborers in the industrial sector, and are also beneficial for rehabilitation purposes (Ebrahimi, 2017).

Supernumerary robotic limbs, on other hand, are additional limbs that augment human capabilities in tasks involving manipulation and locomotion. These can work in conjunction with natural limbs, or operate independently by introducing additional degrees of freedom (DoFs). Some advantages of offering those supernumerary DoFs is the enlarged workspace, as well as the ability to perform sophisticated actions independently that would otherwise be impossible for a single human to perform. Supernumerary robotic legs, for instance, are primarily designed to extend walking abilities (Khazoom et al., 2020) or to offer enhanced stability while standing (Treers et al., 2017). In contrast, supernumerary robotic arms aim to help healthy individuals perform demanding tasks such as construction work (Parietti and Asada, 2014; Ciullo et al., 2019), surgical operations (Abdi et al., 2016), and aircraft fuselage assembly (Parietti and Asada, 2014). Furthermore, supernumerary robotic fingers have been significantly explored due to their ease of attachment and the limited DoFs required for their operation. Various supernumerary robotic fingers have been proposed to assist stroke patients with grasp compensation (Salvietti et al., 2016), offer rehabilitation for hemiparetic upper limbs (Hussain et al., 2017a), and enable healthy individuals in carrying out complex manipulation and grasping tasks (Kieliba et al., 2021).

One of the primary challenges in achieving widespread adoption of WAs is the complexity of their control paradigm. The difficulty arises from the additional overhead attention required by the brain in controlling the WA while maintaining a decent control over the natural limbs (Guggenheim et al., 2020). Conventional implementations of WAs relied on controlling them through joysticks (Nguyen et al., 2019), audio commands (Guo et al., 2022), eye gaze (Fan et al., 2020), sensing parameters related to natural limb's pose (Kojima et al., 2017) and position (Wu and Asada, 2018), or a combination of these (Nguyen et al., 2019). A recent and promising trend is to utilize non-invasive neuro-motor (NM) interfaces, which overcome several issues associated with the previously mentioned conventional methods due to their intuitiveness. This brings us to the importance and functionality of NM interfaces in controlling WAs.

### 1.2 Neuro-motor interfaces

NM interfaces utilize signals such as surface electromyography (sEMG) and electroencephalography (EEG). These interfaces can alleviate the constraint of using natural limbs to control WAs, as is the case when using joysticks.

Several works have demonstrated controlling WAs through sEMG signals. Typically, these utilize sEMG signals from body parts relevant to the desired motion, such as signals from the leg to control a lower body exoskeleton (Chen et al., 2023). On the other hand, EEG-controlled WAs are capable of recognizing user's motion intention through detecting particular cortical activities (Wu and Asada, 2018), in theory making the WA devices more intuitive to control and use. However, EEG-controlled WAs are less common due to the need of sophisticated algorithms to reliably and accurately interpret user intent.

The following subsections provide a background on EEG and sEMG signals. This includes their operational principles, key components of their setups, and the challenges associated with utilizing them as control signals.

#### 1.2.1 Surface electromyography (sEMG)

Electromyography (EMG) is a method that records the electrical activity of muscle cells during contraction (Merletti and Farina, 2016). This activity involves small electrical currents produced by muscle fibers before generating force. These currents result from the exchange of ions across muscle fiber membranes, a crucial part of the muscle contraction signaling process (Day, 2002). sEMG is a non-invasive variant of EMG, where electrodes are placed on the surface of the skin above the muscle of interest. sEMG signals have frequencies lower than 400–500 Hz (Mäki and Ilmoniemi, 2011) and amplitudes in the low mV range (Day, 2002). Notable features of sEMG data exist in time, time-frequency, and frequency domains (Zecca et al., 2002; Rechy-Ramirez and Hu, 2011). Nevertheless, time domain features are the most commonly used for pattern recognition with machine interfaces and WAs in particular (Spiewak et al., 2018).

Generally, there are two primary applications of sEMG in the context of WAs control: sEMG-based motion recognition (Lee et al., 2023) and sEMG-based torque prediction (Lotti et al., 2020). sEMG signals are characterized by a higher signal to noise ratio (SNR) compared to EEG signals. However, for patients who exhibit low sEMG activity or significant muscle spasticity, sEMG-based WAs may not be suitable. In such cases, EEG-based WAs offer a viable alternative.

#### 1.2.2 Electroencephalography (EEG)

EEG is a non-invasive method of capturing electrical cortical activity originating from the simultaneous postsynaptic potentials of neural populations (Cohen, 2017). Electrodes are placed on the scalp to capture the electrical activity associated with cortical brain activation. The amplitude of the measured EEG signals can vary from a few microvolts to several tens of microvolts, influenced by factors such as the individual's mental state, age, and scalp characteristics (Niedermeyer and da Silva, 2005). As EEG signals are small in amplitude, it becomes necessary to amplify those signals for two main reasons. The first is to improve SNR and thus better extract the actual brain signal (Nunez and Srinivasan, 2006). The second reason is to bring the signal to a level suitable for digitization and further processing (Luck, 2014). One notable aspect of EEG data is the neural rhythmic oscillations, where the EEG signals generally have frequencies <100 Hz (Mäki and Ilmoniemi, 2011). Oscillations are split into five main frequency bands: delta (1–4 Hz), theta (4–8 Hz), alpha (8–13 Hz), beta (13–30 Hz), and gamma (30–50 Hz) (Strijkstra et al., 2003). These oscillations are linked to various processes, including sensory, perceptual, cognitive, motor, and emotional functions (Siegel et al., 2012).

EEG data offer two primary types of signals for the control of EEG-based WAs: endogenous, which are voluntarily generated without external stimuli, and exogenous, which are elicited in response to external stimuli. Endogenous signals can be generated when users perform motor imagery (MI) (Pfurtscheller and Neuper, 2001), a process where an individual imagines a movement, leading to observable oscillations in the alpha band at the motor cortex, known as event-related desynchronization (ERD) (Schomer and Da Silva, 2012). Alternatively, endogenous signals can arise from the intention to move, manifesting as an ERD at the motor cortex, or as a movement-related cortical potentials (Wright et al., 2011) that are detectable as a low-frequency signal (Garipelli et al., 2013) at the motor cortex. Endogenous EEG signals provide an intuitive interface for controlling WAs, closely mimicking the way we naturally control our limbs. However, these interfaces often require more extensive training, and the communication bit rate tends to be lower compared to exogenous-based methods (Lee et al., 2017).

Exogenous signals, on the other hand, are evoked by an external stimulus, most commonly through visual cues. This method requires minimal training, and has higher bit rate, however, it occupies the visual modality which is generally needed for performing tasks, thus limiting its applicability. Examples of exogenous signals are steady state visually evoked potential (SSVEP) (Regan, 1966) and P300 (Polich, 2007).

### 1.3 Historical perspective

NM-controlled WAs are typically utilized by individuals who have all their limbs. Interestingly, the technology for these augmentations often originates from innovations aimed at restoring functions for those who have lost one or more limbs, such as prosthetic devices for amputees (Battye et al., 1955; Horn, 1972; Guger et al., 1999; Bitzer and Van Der Smagt, 2006; Ferris et al., 2006; Bai et al., 2015).

**Figure 1:**
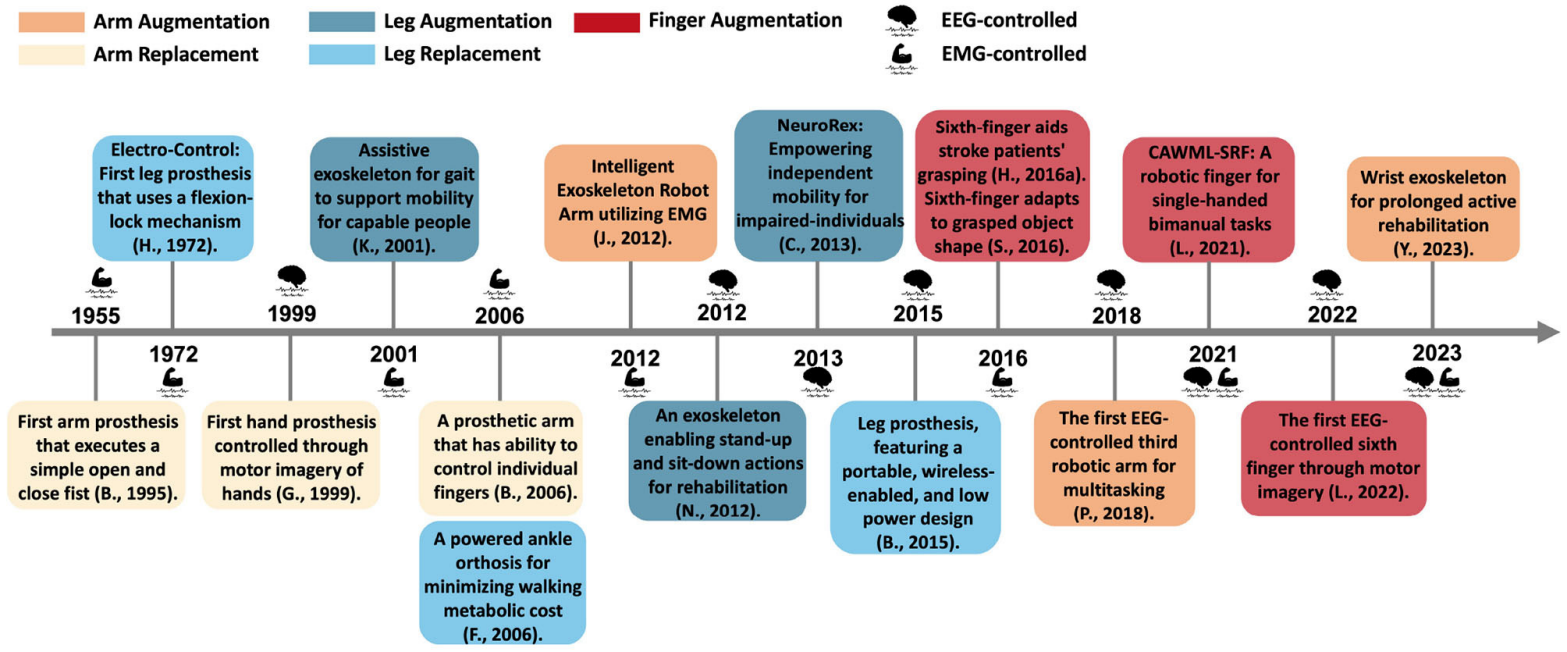
Historical timeline. Significant advancements in wearable technology, designed for either replacement or augmentation, controlled via neuro-motor interfaces, that occurred in the past few decades. Color coding correspond to the type of wearable device and the color-shade indicates whether it is intended for replacement or augmentation. Furthermore, the brain and muscle icons are used to indicate whether the wearable device is controlled through EEG or sEMG interface, respectively.

In fact, the field of prosthetics has historically paved the way for augmentation. Given the intertwined history and shared technologies of the two fields, we have charted a historical timeline highlighting key developments in wearables for both limb augmentation and replacement; see Figure 1. This timeline particularly emphasizes those controlled via sEMG or EEG. The inception of NM-controlled prostheses dates back to 1955 (Battye et al., 1955), when the first hand replacement prosthetic was controlled using sEMG. This innovation evolved to enable basic actions, like opening and closing a fist, using sEMG signals from the forearm. On the other hand, the emergence of EEG-controlled prostheses was not until 1999. That year marked the development of the first hand prosthesis controlled through MI of the right and left hands, specifically to open and close the fist (Guger et al., 1999). Subsequent to this, in 2001, a lower body exoskeleton controlled through four sEMG signals obtained from the thigh was proposed to facilitate the gait cycle of healthy individuals (Kawamoto and Sankai, 2001), marking the first WA controlled through sEMG. A decade later, 2012 witnessed the emergence of the first EEG-controlled WA in the form of a lower body exoskeleton that assisted in pivotal motions, such as transitioning from sitting to standing, with a specific focus on rehabilitation contexts (Noda et al., 2012). The most recent advancement involves augmenting hands with supernumerary robotic fingers controlled via NM interfaces; the first implementation was proposed in 2016 (Hussain et al., 2016b).

Motivated by the inherent intuitiveness of the NM control paradigm and the mounting enthusiasm from the research community, this article offers a comprehensive review of the literature on non-invasive, NM-controlled WAs. Previous reviews have focused on the principles of human movement augmentation and associated challenges (Eden et al., 2022), or on supernumerary robotic limbs, either from a device-driven perspective (Prattichizzo et al., 2021; Yang et al., 2021) or a problem-driven approach (Tong and Liu, 2021). Unlike prior works, our focus is on wearable devices for human augmentation, including supernumerary robotic limbs and exoskeletons controlled exclusively through NM interfaces (either EEG, sEMG, or both). One motivation for reviewing work on EEG/EMG-controlled WAs is our belief that the fusion of both technologies is very promising for achieving reliable augmentation due to the complementary behavior the two modalities could offer.

**Figure 2:**
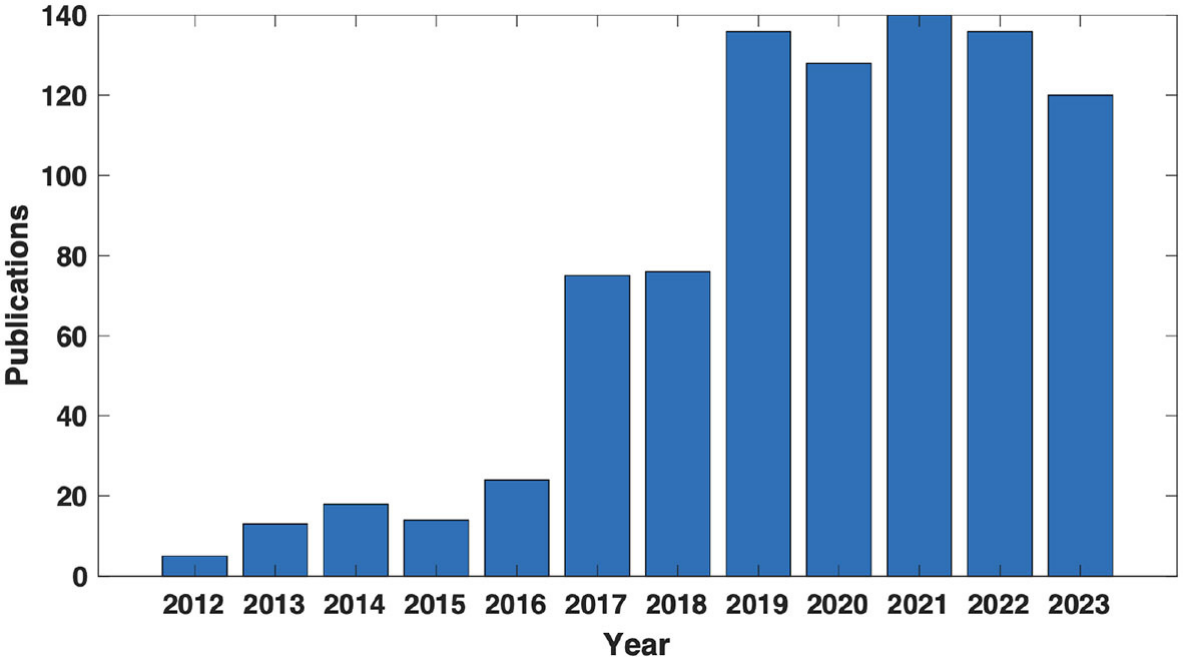
Trend. Annual number of published articles related to wearable augmentations controlled via EEG/EMG, according to the EBSCO database.

We conducted an initial literature search spanning the last twelve years using the following logical combinations of keywords: (EEG OR EMG OR electroencephalography OR electromyography) AND (exoskeleton OR supernumerary) AND (lower body OR upper body OR arm OR hand OR leg OR feet OR foot OR finger). This search was carried out through EBSCO title-abstract-keyword searches. As depicted in Figure 2, the results showcase a clear upward trend in the research work in this domain. Out of these papers, only the relevant ones discussing a physical WA device controlled through EEG, EMG, or both are considered for the segmental analysis. A few other older papers were also considered given their historical importance. Motivated by the inherent intuitiveness of the NM-control paradigm and the mounting enthusiasm from the research community, this article offers a comprehensive review of the literature on NM-controlled wearable robots designed for human augmentation, considering 54 papers in total. All data presented is up-to-date as of September 26th, 2024.

In Section 2, we discuss key concepts related to NM-controlled WAs, including a conceptual model for NM-controlled WAs and the main design considerations for creating functional and user-friendly devices. Additionally, we explore two vital concepts in NM-controlled WAs: the provision of sensory feedback and the neuroplasticity and embodiment associated with using NM-controlled WAs. Section 3 reviews studies that have utilized NM interfaces for WAs, covering supernumerary robotic limbs and exoskeletons from a segmental perspective. Finally, we address some pressing challenges in the field and present trends and future outlooks for NM-controlled WAs.

## 2 NM-controlled WAs: design and integration

### 2.1 Conceptual model

**Figure 3:**
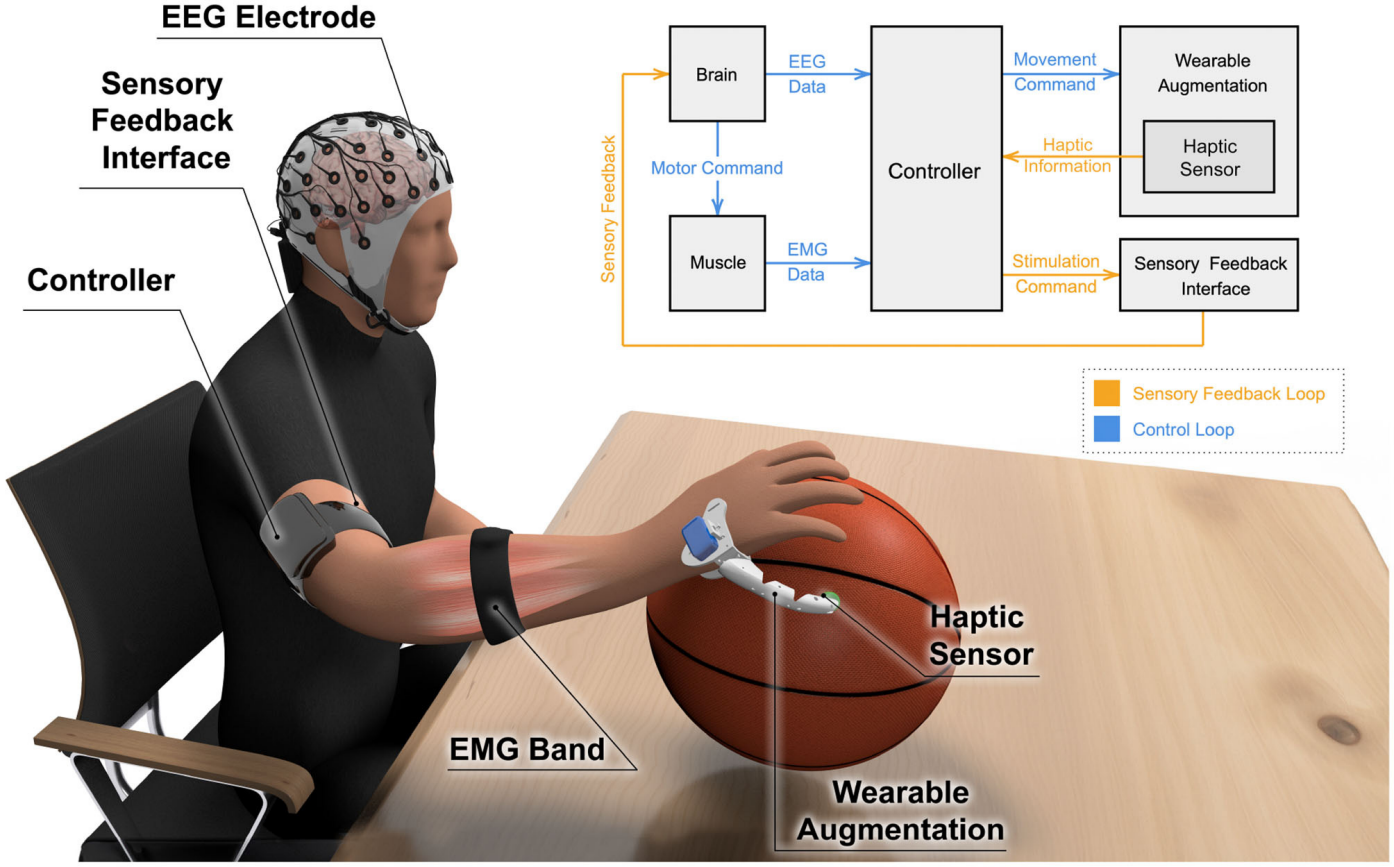
Conceptual model for an NM-controlled WA. This example features a hand WA with a control loop that includes EEG and sEMG to predict movement intent and required force, respectively. The model also includes a feedback loop that conveys sensory feedback to the user.

A conceptual model for an NM-controlled WA is depicted in Figure 3, illustrating a supernumerary finger as a representative example. However, this model is applicable to any WA. It assumes a hybrid control approach, utilizing both EEG and sEMG, although systems may alternatively be controlled solely by one modality. As mentioned earlier, hybrid NM-controlled WAs benefit from the combined features of both modalities. EEG data is used for predicting movement intent, given that EEG is a neural signal that can capture movement intention signals almost instantaneously (Liu et al., 2021), which allows for fast and seamless response. sEMG data, on the other hand, can be used in multiple ways. sEMG from a particular muscle could be a direct means of control to predict the amount of torque or force required (Lotti et al., 2020; Treussart et al., 2020). Another approach is using sEMG from body parts involved with the WA in the task performance, providing context for the WA. This would confine movement possibilities and adjust force or torque to match the collective kinematics of the natural body.

Accordingly, the conceptual model employs EEG and sEMG acquisition systems, where the data acquired from both modalities are transmitted to the WA controller, as illustrated in Figure 3. Haptic sensors attached to the WA, which could measure parameters like contact or grasping forces, transmit sensory data to the WA controller as well. This data is then translated and sent back to the user as either modality-matched or modality-mismatched sensory feedback. After extensive use of the device, it is expected that, due to the phenomenon of brain plasticity, the WA will form its own representation in the brain, fostering a sense of embodiment for the used WA (Kieliba et al., 2021).

### 2.2 Design considerations

**Figure 4:**
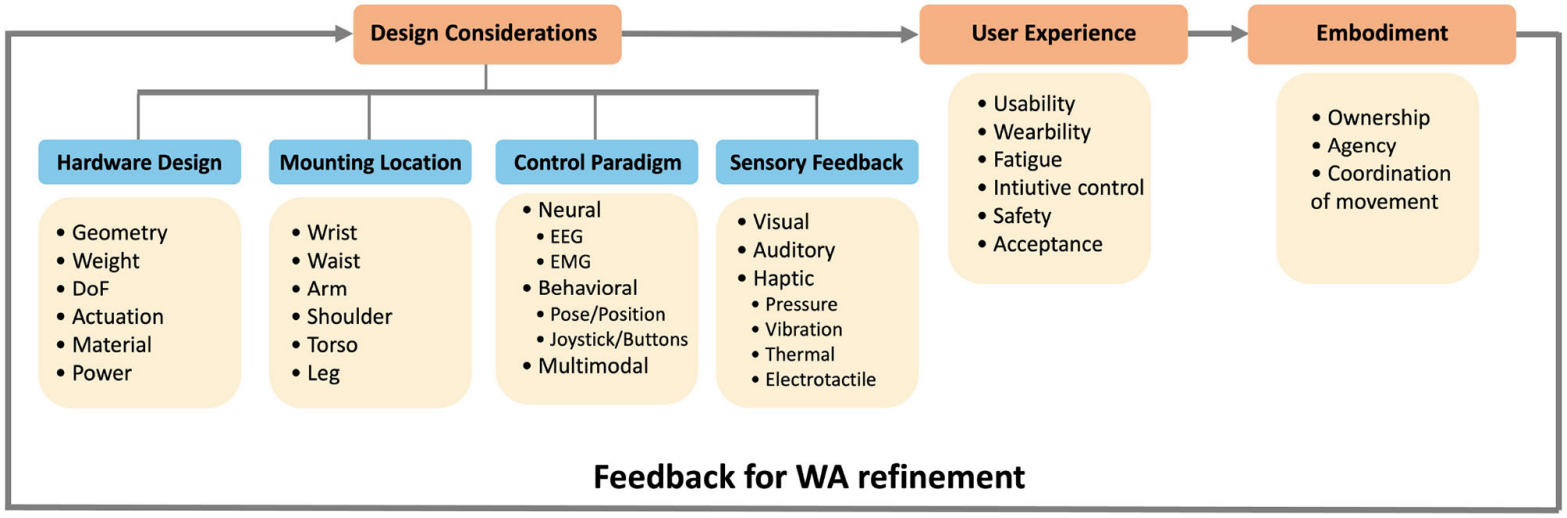
Design considerations of wearable augmentations. Some of these design considerations overlap with those of prostheses; however, the control consideration is unique in this context, as they require employing additional and un-utilized resources of the human body. Furthermore, the mounting locations are more versatile in the case of WAs compared to prostheses. Design considerations have direct impact on the user experience and in the long term, on embodiment.

Designing WAs requires careful consideration of various factors. Our literature review has led us to identify four main considerations essential to the design of WA, as depicted in Figure 4. The first consideration is hardware design, which involves decisions about the augmentation's physical and functional attributes. This includes the WA's shape and structure (geometry), the materials and weight, its range of motion (degrees of freedom), and its sensing and actuation mechanisms. Rigid and fully actuated WAs could provide high precision movements and could reach a variety of configurations but will be heavier in weight, will require larger number of actuators, and could compromise comfort, safety and wearability (Prattichizzo et al., 2021). Soft and under-actuated WAs are lighter and more wearable, but at the cost of reduced functionality. The second consideration is mounting location, which influences the WA's functionality, user comfort, and stability when worn. Some WAs are adjustable and can be installed at several locations of the body. For instance, a supernumerary arm can be installed at the shoulder, or the waist, depending on the intended use (Nguyen et al., 2019), while other WAs are designed to be worn at a specific body location, such as a lower-body exoskeleton used to support gait (Kawamoto and Sankai, 2001). The third consideration is the control paradigm. This component, arguably one of the most challenging in design, is concerned with interfacing the WA with its user, thereby granting them agency over the WA's movements. In fact, the control consideration presents unique challenges for WAs from both conceptual and practical perspectives. There are many control interfaces that have been proposed in the literature, such as using joysticks, non-physiological human body measurements (contact forces, limb pose, limb position), and physiological human body measurements (EEG, sEMG). The fourth consideration is the provision of sensory feedback to the user through visual, auditory, or haptic feedback. For supernumerary robotic limbs, this can mean feedback on grasp strength, stride force, or the point of contact. For exoskeletons, sensory feedback might convey information such as mediolateral and anteroposterior weight shifts (for lower body exoskeletons), as detailed in Muijzer-Witteveen et al. (2018).

Design choices made during the development of a WA directly impact the user experience, both when the device is worn and when in use. Usability, encompassing ease of use and learnability, is influenced by several factors, including control and sensory feedback mechanisms. Wearability, related to the user's comfort and thus affecting how long the device can be worn, is shaped by choices regarding the mounting location, weight, and material of the WA. Fatigue, closely linked to wearability, addresses both the physical and mental impact of long-term WA use, such as muscular and cognitive fatigue from ongoing interaction or sensory overload. The intuitiveness of control is crucial as it should facilitate coordination with the user's movements. Safety is another key aspect, ensuring the WA poses no physical danger or compromises user data security. Lastly, acceptance, influenced by social and psychological factors, plays a critical role in the WA's adoption.

After extended usage, user experience has a direct influence on embodiment, including the sense of ownership, agency, and the ability to coordinate the WA's movement with the rest of the body. Users providing feedback on their sense of embodiment can feed back into the design process, potentially leading to modifications in the WA's hardware, control, or sensory feedback mechanisms. These improvements could lead to a version that fosters better embodiment.

### 2.3 Sensory feedback

Sensory feedback, one of the design considerations, is defined as the provision of cues to users about the interaction between the WA, the human body, and the environment (Pinardi et al., 2023). This can lead to enhanced control and motor functionality (Clemente et al., 2019; Zollo et al., 2019), as well as increased sense of embodiment (Di Pino et al., 2020; Page et al., 2018). As stated in previous literature, there are three main considerations when designing a sensory feedback mechanism for a WA: the type of interaction information that needs to be conveyed (measured parameter), the form in which it is delivered to the user (feedback modality), and a feedback assessment mechanism. The measured parameter required depends on the WA's function. For example, parameters such as contact force, contact location, position, velocity, acceleration, temperature, and joint angles are relevant for supernumerary limbs (Pinardi et al., 2023), whereas weight-shift and force exertion are pertinent for lower and upper body exoskeletons, respectively. Such parameters can be conveyed to the user through modality-matched (e.g. displaying pressure information using a pressure actuator) or modality-mismatched (e.g. displaying pressure information using visual feedback) stimulation. As explained in Figure 4, feedback modalities can include visual, auditory, or haptic (including both, tactile and proprioceptive), each with its own pros and cons. As for the sensory feedback assessment, this is generally measured through four main metrics: accuracy while using the WA, latency between the event and perceiving the sensory feedback, task completion time (Shadmehr and Mussa-Ivaldi, 2012), and force regulation. Readers interested in sensory feedback for human augmentation are directed to the work by Pinardi et al., where this topic is discussed extensively.

Sensory feedback in NM-controlled WAs presents unique challenges and requires additional considerations compared to augmentations controlled by other methods. One critical aspect is the timely delivery of sensory feedback, irrespective of its type. While prompt feedback is crucial for all WAs to ensure seamless control (Cipriani et al., 2014; Sensinger and Dosen, 2020), it is especially vital for NM-controlled WAs. These devices require cognitive training prior to their use; thus, any delay in feedback can significantly affect the learning process and the effective control of the WA. In certain implementations, it is necessary to provide users with feedback upon the detection of EEG/sEMG signals that control the device (Franco et al., 2019; Cisnal et al., 2023). This notification is delivered to the participant before they can observe the resulting physical movement of the wearable augmentation. Such proactive feedback is designed to alleviate any user anxiety regarding their ability to produce the correct EEG/sEMG signals. Consequently, any delay in this feedback also presents a significant challenge, as it can affect user confidence and the overall effectiveness of the device.

Given that NM-controlled WAs require cognitive effort toward their control, additional mindfulness is required while designing the sensory feedback mechanism used. Particularly, a careful design is required in choosing the appropriate tradeoff between noticeability and frequency of the feedback, for an optimized cognitive load. We deem an adjustable sensory feedback mechanism is necessary for NM-controlled WAs to tackle this trade-off. Tailoring the type, intensity, and timing of the feedback to the user's preferences and needs could help achieve this. Also, sensory feedback could be adapted based on the users' performance, by providing more pronounced feedback for situations where users fail to perform the task properly. As users gain proficiency, the frequency of sensory feedback could be reduced to decrease cognitive burden and enhance usability.

### 2.4 Embodiment and neuroplasticity

A desired outcome of sensory feedback for an augmentation is embodiment, which often is associated with improved performance and utility. This means that the device is perceived as either part of, or an extension of, the body. Embodiment can be self-reported, but from a neurological standpoint, it is particularly important in the context of NM-controlled WAs. This form of embodiment can be validated by examining neurophysiological signals related to the movement of the WA and those of the natural limbs. For example, in EEG, a distinct and repeatable neural activation, in the form of an ERD, may occur during MI of the augmented device, indicating embodiment. Similarly, in sEMG-controlled WAs, initial attempts may result in noisy sEMG signals, but as the user achieves embodiment, these signals become more standardized and less noisy. These neurophysiological changes suggest neuroplastic adaptations in the brain.

Neuroplasticity is the ability of the brain to adapt its structure and functional connectivity in response to novel external conditions that promote new functions or reorganize old ones (Di Pino et al., 2014). This is the neural basis of early development in children (Hensch, 2005), acquisition of new skills (Pascual-Leone et al., 1995), and recovery from brain injuries (Chen et al., 2002). The process itself is gradual, with three phases of acquiring a new representation in the brain: the initial habituation, consolidation, and long-lasting plasticity (Karni et al., 1998). In the context of NM-controlled WAs, persistent and prolonged usage of a WA device is anticipated to induce neural changes in the motor cortex due to the motor control of the WA. Furthermore, depending on the sensory feedback modality, neuroplastic changes could occur in the somatosensory cortex (haptic feedback), visual cortex (visual feedback), and auditory cortex (auditory feedback). This is inspired from previous literature on neural plasticity of biological limbs, where it was reported that expert tennis players had enlarged hand representation in the motor cortex (Pearce et al., 2000), while violin players had enlarged somatosensory representation of their fingers (Elbert et al., 1995).

## 3 NM-controlled WAs: segmental perspective

This section presents a comprehensive review of NM-controlled WAs found in the literature and categorizes them based on their functionality. The first category is hand WAs consisting of devices that are designed to augment hand-related functions. These could be in the form of supernumerary fingers or hand/wrist orthoses. The second category is upper body WAs in the form of supernumerary arms and upper body exoskeletons. These wearable devices are concerned with providing support to the upper body in performing gross manipulation tasks and increasing endurance during industrial tasks. The third category is lower body WAs in the form of supernumerary legs and lower body exoskeletons. These devices are concerned with providing extended stability and standing support, assisting in restoring locomotion, or reducing the metabolic cost of gait.

### 3.1 Hand wearable augmentations

Hand WAs come in various forms, including hand orthoses that assist stroke patients and supernumerary fingers that serve as assistive aids or augmentative tools. These devices are crucial for compensating for the loss of hand abilities caused by strokes, offering solutions to regain grasping capabilities. For healthy individuals, they enhance hand dexterity for complex tasks and expand the manipulation workspace. Hussain et al. have been at the forefront of hand WAs research since their pioneering work on supernumerary fingers commenced in 2014. Their earliest implementation of a hand WA was motion-controlled (and not NM-controlled), where hand movements recorded by a smart glove were translated into commands to control the supernumerary finger, aimed to enhance manipulation dexterity (Prattichizzo et al., 2014). The first sEMG-controlled hand WA was introduced in 2016 in the form of a supernumerary finger (Hussain et al., 2016a), targeting stroke patients. The developed supernumerary finger acted as one part of a gripper, while the paretic hand as the other part of the gripper, compensating for the lost ability to grasp. The supernumerary finger was controlled through an eCap interface (with three electrodes) that utilized sEMG signals from the frontalis muscle for its flexion and extension. The control mechanism was based on a finite state machine, where one frontalis muscle contraction could move or stop the movement of the finger in a particular direction, while two consecutive contractions switched the control direction from flex to extend or vice versa. The functionality and effectiveness of the supernumerary finger were tested with six stroke patients who went under the Frenchay Arm Test. Patients were able to perform some of the Frenchay Arm Test tasks with the supernumerary finger such as grasping a cylinder and picking up a glass, which they could not perform without the supernumerary finger. However, some patients failed performing some of the Frenchay Arm Test tasks despite using the supernumerary finger (Hussain et al., 2016a); those failures were caused by the limited arm mobility. Following the same control mechanism, authors equipped the sEMG-based supernumerary finger with an arm support system used during the rehabilitation period (Hussain et al., 2017a). The proposed system enabled stroke patients to perform some of the Frenchay Arm Test tasks that they could not perform with the supernumerary finger alone, such as combing their hair.

To enable precise manipulative tasks, a sEMG-based supernumerary finger capable of performing both a precision and power grasp, involving the fingertip or the full finger respectively, was developed (Salvietti et al., 2016). The flexion and extension control mechanism was based on the frontalis muscles, similar to that described by Hussain et al. (2016a), but included an additional control signal based on three muscle contractions, allowing switching between power and precision modes. In another study (Hussain et al., 2016b), the issue of compliance of the supernumerary finger was addressed using a dual sEMG interface. The first component consisted of a sEMG armband placed on the forearm, which recognized five different gestures and associated them with movements of the supernumerary finger. For instance, “wave-in” and “wave-out” gestures corresponded to the respective extension and flexion of the supernumerary finger, while a “closed-fist” gesture triggered full extension, and “fingers spread” signaled a stop. The other sEMG interface was located at the bicep of the contralateral arm and continuously measured compliance, specifically the tightness of the grip. This prototype was tested on both impaired and healthy individuals; results showed that users were able to complete the desired tasks for compensation and augmentation with the supernumerary finger successfully.

In 2017, an enhanced sEMG-controlled hand WA prototype with multiple fingers was proposed (Hussain et al., 2017b). Such augmentation with higher DoFs is more suitable for high payload tasks. This work employed the same control mechanism as presented by Hussain et al., but enhanced the control by incorporating auto-tuning calibration to better accommodate the user-dependent nature of the sEMG signals. Similarly, Leigh and Maes (2016) developed a wearable sEMG-based multi-joint interface with two flexible, finger-like extensions. Beyond grasping, the proposed augmentation could carry objects or turn a doorknob while hands are occupied, and could provide a stable base or support for writing. Those were controlled through a sEMG-based gesture recognition through an armband placed at forearm close to the elbow.

Seeking a more natural interface, Liu et al. (2021) proposed a flexible supernumerary finger controlled by a hybrid EEG-sEMG system. sEMG signals were measured from the frontalis muscles, while EEG signals were measured from the contralateral motor cortex. A 4-week study involving 10 healthy subjects revealed that employing EEG to initiate grasping and sEMG to release objects led to faster task completion (~7 s) and higher success rates (~90%), specifically in bimanual tasks performed with one hand. Grasp initiation was triggered through a detection of ERD at C3 electrode as a result of performing the MI of flexing the supernumerary finger; this detection was done through a trained convolutional neural network. sEMG signals from eyebrow raises, processed with a simple threshold method, controlled object release. A follow up study attempted to use exclusively EEG for control using MI (Liu et al., 2022). A convolutional neural network model was used for identifying MI of the supernumerary finger's flexing. A genetic algorithm was used to select optimal channels for controlling the supernumerary finger, thus enhancing adaptability for individual users. A real-time control experiment was undertaken with 10 subjects, which involved bending the supernumerary finger; an average classification accuracy of 70% was achieved. No mechanism was proposed for the extension of the finger in this work.

Several supernumerary robotic fingers implementations utilized sensory feedback in their design to improve control and embodiment of the WA. Meraz et al. (2017) developed a sEMG-controlled supernumerary finger where differential electrodes located behind the ear were used to capture the sEMG signal and to control the finger's fingertip in two directions: horizontally and vertically. The system was equipped with dual feedback mechanisms: visual and haptic. The visual feedback was provided via a head-mounted display that showed the finger's position, whereas the haptic feedback was conveyed through electrical stimulation on the palm, signaling contact with external objects. In subsequent work, Shikida et al. (2017) developed a sEMG-controlled supernumerary finger operated by signals from the left and right posterior auricular muscles. The bending angle of the thumb was determined by the activation level of these muscles, with the robotic finger moving toward the side of the stronger muscle contraction. The WA was designed to provide sensory feedback about the joint angle of the thumb through various vibration patterns delivered at the bases of the index and pinky fingers. This feedback was found to increase the operability of the supernumerary finger through the observed reduction in mistakes in a finger-reaching task. Another work that utilized the same control mechanism for the supernumerary finger (Aoyama et al., 2019) employed an alternate sensory feedback method that relies on the vibrotactile phantom sensation. Using two vibration motors positioned at the back of the palm, the intensity of vibration for each motor varied depending on the position of the supernumerary finger. This variation altered the perception point accordingly, which corresponded to the finger position. Another work (Franco et al., 2019) proposed the use of vibrotactile feedback for sEMG-controlled supernumerary finger to solve a very relevant problem; correctly detecting the intent of moving the supernumerary finger through sEMG. Conventionally, subjects have to wait to see the impact of their control visually which is time consuming and impractical. Authors found that providing vibrotactile stimulation on the forehead, signaling a detection of a sEMG sourced command, reduced the required muscle effort and the time needed to perform tasks.

As for hand orthoses, most of the implementations are developed to aid in the rehabilitation of wrist and finger functions for stroke patient. Many of these devices utilize sEMG-based implementations. In such setups, sEMG signatures corresponding to rehabilitation movements, such as opening, closing, relaxing, or forming a spherical grip with the hand, are captured. The mapping of the sEMG signals to these specific movements is achieved through various methods such as simple thresholding (Cisnal et al., 2023), machine learning classifiers (Park et al., 2020), or deep learning models (Chen et al., 2021). As for EEG-based orthoses, an implementation by Araujo et al. utilized MI of the right and left hand to respectively control the flexion or cessation of flexion in all fingers. However, there was no mention of a mechanism for extension. After a training session, an LDA classifier was employed to differentiate between the two classes. In 2023, a novel wrist orthosis utilizing both sEMG and EEG control was developed to facilitate six distinct gestures (Yang et al., 2023). The system primarily operated on sEMG control, acquired through an armband and processed by a convolutional neural network to map sEMG patterns to the specific gestures. However, when fatigue was detected, the system automatically switched to EEG control to prevent further fatigue. EEG signals were acquired using three electrodes placed on the forehead, and control was binary–indicating either the presence or absence of motion intent. The potential movements were displayed on a screen, and the subject decided whether to ignore the cue or intend to move. A summary of the hand WA literature along with their specifications is shown in Table 1.

**Table 1:** Specifications summary of hand WAs.

**Device name**	**Control modality**	**Measurement site**	**Target population**	**Attachment**	**Year**
Soft-SixthFinger (Hussain et al., 2016a)	sEMG	Frontalis muscle	Chronic stroke patients	Wrist-mounted	2016
Supernumerary robotic finger (Hussain et al., 2016b)	sEMG	Bicep muscle	Chronic stroke patients and healthy subjects	Wrist-mounted	2016
Robotic sixth finger (Salvietti et al., 2016)	sEMG	Frontalis muscle	Chronic stroke patients	Wrist-mounted	2016
Programmable joints interface (Leigh and Maes, 2016)	sEMG	Brachioradialis muscles	Healthy subjects	Wrist-mounted	2016
Soft supernumerary robotic Finger (Hussain et al., 2017a)	sEMG	Frontalis muscle	Post stroke patients	Wrist-mounted	2017
Robotic extra thumb (RET) (Meraz et al., 2017)	sEMG	Frontalis muscle	Healthy subjects	Wrist-mounted	2017
Soft sixth finger and double soft sixth finger (Hussain et al., 2017b)	sEMG	Frontalis muscle	Chronic stroke patients	Wrist-mounted	2017
Extra robotic thumb (ERT) (Shikida et al., 2017)	sEMG	Posterior auricular muscles	Healthy subjects	Attached on the left hand by two belts	2017
sEMG-based robotic thumb (Aoyama et al., 2019)	sEMG	Posterior auricular muscles	Healthy subjects	Attached on the left hand by two belts	2019
sEMG-based robotic sixth finger (Franco et al., 2019)	sEMG	Frontalis muscle	Chronic stroke patients	Worn on the paretic forearm	2019
A wearable hand robot (Park et al., 2020)	sEMG	Forearm surface	Chronic stroke patients	Placed on forearm and fingers	2020
Soft exoskeleton Glove (SExoG) (Chen et al., 2021)	sEMG	4 electrodes at the forearm	Chronic stroke patients	Worn on the paretic hand	2021
RobHand exoskeleton (Cisnal et al., 2023)	sEMG	Extensor and flexor digitorum	Chronic stroke patients	Worn on the paretic hand	2023
HERO (hand orthosis) (Araujo et al., 2021)	EEG	16 channels (10-20 system)	Chronic stroke patients	Worn on the paretic hand	2021
MI-based sixth finger (Liu et al., 2022)	EEG	Parietal and frontal cortex	Healthy subjects and stroke patients	Wrist-mounted	2022
Modular and wearable supernumerary robotic finger (Liu et al., 2021)	sEMG; EEG	Frontalis muscle; Central cortex	Patients with hand motor function impairment and healthy subjects	Wrist-mounted	2021
Wrist exoskeleton (Yang et al., 2023)	sEMG; EEG	8 channels at the forearm; Prefrontal cortex	Stroke patients	Wrist-mounted	2023

### 3.2 Upper body wearable augmentations

Upper body exoskeleton and supernumerary arms both serve to enhance upper body functions, yet they are tailored for distinct applications. Upper body exoskeleton serve a dual purpose. They are essential for the rehabilitation and functional restoration of individuals with upper limb impairments. Additionally, they provide robust support for healthy individuals, cushioning shock during drilling tasks, enhancing endurance for extended weight carrying, and facilitating the lifting of heavy loads for brief duration. Conversely, supernumerary arms are designed to broaden the workspace and increase the DoF for able-bodied users, or to serve as a substitute arm for individuals with impairments, such as stroke patients, offering an alternative to the affected limb.

The vast majority of upper body exoskeletons found in the literature are controlled through sEMG. Focusing on those designed to augment the abilities of healthy individuals, Zhang et al. (2019) developed a sEMG-based upper body exoskeleton that enhanced bicep function. A closed-loop system measured bicep activity via sEMG sensors and adjusted a pneumatic muscle's pressure to match the exertion, allowing users to control their force output. This exoskeleton doubled the lifting capacity for industrial workers, allowing them to perform repetitive lifting tasks with twice the efficiency. Another example is by Lotti et al. (2020) who developed a real-time sEMG-based upper body exoskeleton for elbow support. This exoskeleton used three sEMG channels and the joint angle of the elbow to calculate the elbow flexion-extension torque, where a calibration session was conducted for the controller taking individual anthropometric features into consideration. Using this exoskeleton significantly reduced exerted force by the users across different loads up to 2 kg. A subsequent study (Treussart et al., 2020) developed a sEMG-based control mechanism for a rigid upper body exoskeleton with the goal of assisting biceps with carrying unknown loads. The system was able to predict the direction and intensity of the intended movement and was able to adapt to different users through calibrating user-specific parameters in the control method. The controller used a closed-form non-linear exponential-power relationship to estimate torque from the sEMG signal. A significant reduction of physical strain and muscles effort was observed upon using the exoskeleton device. Recently, Lee et al. (2023) developed a soft upper body exoskeleton to provide further strength to multiple joints in the shoulder and elbow using pneumatic artificial muscles. The system operated by interpreting sEMG signals from the shoulder and upper arm to control the exoskeleton, with a cloud-based artificial intelligence solution predicting the user's movement intentions. The WA could differentiate between four intended movements (shoulder/elbow, flexion/extension) with an average accuracy of 96.2%, offering four times the strength to the user, and featuring a rapid response rate of <250 ms.

Similarly, upper body exoskeletons used for rehabilitation are predominantly controlled through sEMG. An early implementation was proposed by Kiguchi and Hayashi (2012), a sEMG-based impedance control method for a rigid upper body exoskeleton for self-rehabilitation practices. The algorithm dynamically controlled the WA's stiffness and damping properties, adapting to the user's unique sEMG signal features and specific body characteristics through a neuro-fuzzy modifier. Another study employed two sEMG channels on the biceps and triceps to assist in carrying loads up to 10 kg. In this work, the sEMG amplitude was mapped to a force exertion by the upper body exoskeleton using a fuzzy logic algorithm (Jeon et al., 2012). A subsequent work (McDonald et al., 2017) proposed a sEMG-based rehabilitation upper body exoskeleton system for spinal cord injury (SCI) patients. The system was designed to predict an intended movement out of sixteen possible movement involving the wrist, elbow, and forearm from the sEMG waveforms. Linear discriminant analysis was used to classify the intended movements of the users, involving single or multiple degrees of freedom or a combination of both. A classification sensitivity of 82% and 66% were achieved for the healthy population and the SCI patients, respectively.

A unique implementation of a rehabilitation upper body exoskeleton was by Kawase et al. (2017) where a hybrid EEG/sEMG control system was utilized for real-time rehabilitation and support. The desired motion was selected using EEG signals through an SSVEP paradigm and an SVM classifier, which relied on features extracted from a single electrode at the occipital cortex. Meanwhile, sEMG signals were utilized to predict the intended joint angle; this estimation was performed using a mathematical model of the musculoskeletal system commonly applied in neuroscience research (Maintained, 2009). Their experiments with SCI patients demonstrated the exoskeleton's ability to assist users in carrying objects effectively. Particularly, users positioned their arms at appropriate angles, with a correlation coefficient between the required and measured angles of 0.9 for SCI patients.

Unlike upper body exoskeletons, the supernumerary arms are relatively recent. The first NM-controlled supernumerary arm was proposed in 2018 by Penaloza and Nishio (2018) and Penaloza et al. (2018). An EEG-controlled supernumerary arm for multitasking side-by-side with the natural arms through MI was demonstrated. Authors also explored equipping the supernumerary arm with vision capabilities to evaluate the context of the manipulation task (Penaloza et al., 2018). A calibration session was conducted for each subject to optimize the EEG frequency band and channels, setting a simple threshold on the PSD values to distinguish between grasp and release actions. The EEG signal was used to detect the intention of the grasp while the camera was used to detect the object thus choosing a suitable grasping method. The supernumerary arm demonstrated successful object grasping and releasing capabilities, distinguishing between grasp and release actions with an average accuracy of 70% during multitasking. A year after, a novel soft supernumerary arm akin to an elephant trunk was proposed, with reconfigurable end effectors based on the task (suction, grasper, and holder) (Nguyen et al., 2019). The proposed supernumerary arm used pneumatic actuation and was mountable at different locations. Two sEMG signals acquired from both biceps were used to control the pressurization (three levels) and the direction of the motion (8 angles), respectively, based on simple threshold mapping. Test scenarios such as opening a door, picking an object, and holding an umbrella were carried out, and the supernumerary arm demonstrated its ability to carry up to 3.8 kg. Recently, Tang et al. (2022) proposed an EEG-based supernumerary arm with three rigid fingers, developed for stroke patients with a nonfunctional natural arm. The system was comprised of a module for grasp intention recognition using EEG data through MI or natural limbs and another module for object detection to identify the position of the target. The outputs of both modules fed into a hybrid module for arm trajectory estimation. Graph convolutional networks and gated recurrent unit network models were used for EEG intent predictions, leading to an average grasping success rate of 86.44%.

Apart from developing supernumerary arms for enhancement or rehabilitation purposes, Asada's team developed two, stick-like, supernumerary arms controlled through abdominal muscles, to explore the dynamics of using both natural and supernumerary arms concurrently (Guggenheim et al., 2020). Users were requested to minimize the positional discrepancy between each of the four limb's tip and specific targets (Guggenheim et al., 2020). It was found that during multitasking, participants used their natural arms first before using their supernumerary arms with a significantly different movement starting time. Such a finding suggests giving priority for natural limbs for time-sensitive tasks. A summary of the upper body WA literature along with their specifications is shown in Table 2.

**Table 2:** Specifications summary of upper body WAs.

**Device name**	**Control modality**	**Measurement site**	**Target population**	**Attachment**	**Year**
Intelligent exoskeleton robot arm (Jeon et al., 2012)	sEMG	Biceps and tricep muscles	Individuals with disabilities	Forearm	2012
Upper body power-assist exoskeleton (Kiguchi and Hayashi, 2012)	sEMG	16 muscles spanning the arm, chest and shoulder	Healthy subjects	Upper arm and forearm	2012
MAHI Exo-II (McDonald et al., 2017)	sEMG	8 muscles including the biceps, triceps and forearm muscles	Spinal cord injury patients	Forearm	2017
Soft poly-limbs (Nguyen et al., 2019)	sEMG	Biceps brachii muscles	Impaired and healthy subjects	Placed on a shoulder and waist harness	2019
Soft robotic bicep augmentation (Zhang et al., 2019)	sEMG	Biceps brachii muscles	Industrial workers	Attached to the arm with a shoulder harness	2019
Supernumerary limbs during independent tasks (Guggenheim et al., 2020)	sEMG	Pectoralis major and rectus abdominis muscles	Healthy subjects	Waist-mounted	2020
Soft wearable arm exosuit (Lotti et al., 2020)	sEMG	Bicep, tricep, and brachioradialis muscles	Elbow support for healthy subjects	Attached with a shoulder harness	2020
Upper body exoskeleton for carrying Unknown Load (Treussart et al., 2020)	sEMG	Bicep and tricep muscles	Healthy subjects	Upper arm and forearm	2020
Intelligent upper body exoskeleton (Lee et al., 2023)	sEMG	Biceps and triceps brachii, and medial deltoid muscles	Elderly	Attached to a backpack to support shoulder and elbow	2023
BMI of a third arm for multitasking (Penaloza and Nishio, 2018)	EEG	Frontal and parietal cortex	Healthy subjects	Next to the user	2018
Human-like robotic limb (Penaloza et al., 2018)	EEG	Contralateral motor cortex	Healthy subjects	Next to the user	2018
Wearable supernumerary robotic limb system (Tang et al., 2022)	EEG	64 channels (10–20 system)	People with upper-limb motor disorder	Mounted on users' right shoulder with an elastic strap	2022
Whole-arm exoskeleton (Catalán et al., 2023)	EEG	5 channels at the contralateral hemisphere	Impaired individuals	Upper arm and forearm	2023
A hybrid BMI-based exoskeleton (Kawase et al., 2017)	sEMG; EEG	8 muscles including the biceps, triceps, forearm muscles; occipital cortex	Paralyzed individuals	Worn as a vest with arm attachments	2017

### 3.3 Lower body wearable augmentations

Lower body exoskeletons and supernumerary legs both fall under lower body WAs. Lower body exoskeletons are versatile devices designed to address various mobility needs including gait support for the elderly or individuals with walking disorders. For those with paraplegic conditions, lower body exoskeletons enable upright walking and thus granting independence. Additionally, they play a pivotal role in the rehabilitation and restoration of walking abilities for individuals recovering from SCI or strokes (Contreras-Vidal and Grossman, 2013), facilitating the retraining of muscles and neural pathways through repetitive motion and therapeutic exercises.

Several commercially available lower body WAs, in the form of exoskeletons, have been utilized in research literature, where NM-control paradigms are developed for their operation such as Rex (Rex Bionics, 2023), Lokomat (Hocoma, 2023), and HAL (Lower Limb HAL, 2023). For unique research goals or to explore innovative control strategies, some research groups have developed their own custom-made lower body exoskeletons.

The first NM-controlled lower body exoskeleton, HAL-3, was developed in 2001 (Kawamoto and Sankai, 2001); earlier versions of this WA were controlled through other non-physiological means. HAL-3 was controlled by sEMG signals from the upper leg and designed to offer power assistance, aiding in ambulation for patients who could move their muscles but require additional strength for movement. The WA predicted joint torque based on the acquired sEMG parameters through a linear equation where its parameters were calibrated for each subject. To mitigate any discomfort from inaccurate torque predictions or time delays, the system incorporated a feedforward controller, ensuring rapid response for power assistance.

A limitation of sEMG-based lower body exoskeleton is their unsuitability for stroke patients with low sEMG activity or significant muscle spasticity. An alternative is EEG-based lower body exoskeletons, where such implementations are based either on endogenous or exogenous signals (see section 1.2.2). The first EEG-controlled lower body exoskeleton was proposed in 2012, called EEG-oneDoF, due to its single DoF designed to assist with stand-up and sit-down movements (Noda et al., 2012). This custom-made WA was controlled through MI of right- and left-hand movement; EEG data was formed into a covariance matrix and fed into a trained linear classifier achieving an accuracy of 71%. Since the output of the classifier can fluctuate, a hysteresis algorithm was employed. The EEG-oneDoF exoskeleton, was equipped with visual feedback. Visual feedback was displayed on a screen showing the probability of the EEG decoder predicting stand-up or sit-down.

Similarly, Do et al. proposed a predictive model that detects kinesthetic MI of walking, aiming to enable gait for patients with SCI. EEG data segments of 0.75 s were acquired every 0.25 s using a sliding overlapping window. A state transition was guaranteed when the model consistently predicted that state for 2 s, achieving an accuracy of 86.3% (Do et al., 2013). Another study (Lee et al., 2017) introduced a cascaded classification system that differentiates between walking and turning movements using EEG data. Users performed MI tasks involving either moving or relaxing both hands. These MI signals controlled the first stage to predict walking versus turning, and the same movements differentiated turning right from turning left in the subsequent stage. ERD data from the motor cortex were utilized for prediction, with a random forest classifier handling classification. The proposed system achieved an average accuracy of ~92.4%. Real-time visual feedback was displayed on a screen, showing the action predicted from brain signals and the subsequent actions to be performed by the WA.

Other studies have also explored the concept of intent prediction, the other form of endogenous control, applied to lower body exoskeleton. For instance, Kilicarslan et al. developed Neuro-Rex, a lower body exoskeleton designed to facilitate walking, turning, sitting, and standing for paraplegic patients. This was done by predicting intent from movement-related cortical potential below 2 Hz (Kilicarslan et al., 2013). The system achieved a three-class accuracy of 97% for walking, turning right, and turning left motions and 99% for sitting, resting, and standing motions. Building on this, further research introduced an EEG-based intent detection system which aided gait in stroke patients using the Lokomat Pro, a commercialized lower body exoskeleton tailored for such applications (Garćıa-Cossio et al., 2015). Employing a logistic regression classifier and focusing on alpha and beta bands at the sensorimotor cortex, the system achieved 89% accuracy in predicting walking and idling states. A similar lower body exoskeleton was proposed to enable gait, but based on both MI and movement intent (López-Larraz et al., 2016). The system was able to achieve 84 and 70% decoding accuracy for healthy and SCI patients, respectively, in choosing between rest and walk conditions.

Lower body exoskeletons have also been successfully controlled by exogenous EEG signals, predominantly using SSVEP. For example, Kwak et al. (2015) made use of five flickering LEDs at unique frequencies from 6 to 13 Hz to elicit SSVEPs detectable in the occipital cortex. These potentials were then classified using k-nearest neighbors classifier and utilized as control signals to command the lower body exoskeleton. These commands were walking forward, turning right, turning left, sitting, and standing, and achieved an accuracy of 91%. Another innovative study (Gui et al., 2017) introduced a locomotion trainer with multiple gait patterns, which operated on a hybrid control approach employing both EEG and sEMG signals. This system used four flickering LEDs at different frequencies to trigger SSVEP, corresponding to four control modes: stop, walk, speed up, and slow down. Concurrently, sEMG signals from the right knee joint were analyzed to predict the torque required for the task. This dual-modality system was tested on both healthy individuals and stroke patients, achieving an accuracy of ~ 92.40% in detecting the four movements using a LDA classifier.

Apart from using lower body WA for locomotion and gait assistance, EEG-controlled systems have been used for neuro-rehabilitation purposes, creating tangible impact on patients neural pathways. Such investigation was conducted by Donati et al. (2016), where eight individuals with chronic SCI underwent training for a year. The paradigm incorporated immersive virtual reality training using brain signals to control a virtual avatar and later to control a custom-made lower body exoskeleton with tactile feedback. This formed a feedback loop enabling real-time observation of brain activity outcomes. Results indicated that all eight patients exhibited neurological enhancements in somatic sensation and enhanced ERD during MI. As a result, 50% of these patients were upgraded from chronic to incomplete paraplegia classification. For sensory feedback, the work employed force sensors on the lower body WA and a multi-channel haptic display (vibrators) on the patients' forearms to provide continuous tactile feedback during gait training. The results showed that the feedback mechanisms may contribute to cortical and subcortical plasticity, potentially aiding in partial neurological recovery.

Beyond rehabilitation and gait assistance, research explored the use of lower body exoskeleton for reducing the metabolic cost of walking for healthy individuals. One study (Gordon and Ferris, 2007) investigated a pneumatically powered ankle exoskeleton that operated via sEMG control, where the exoskeleton's activation was proportional to the user's soleus sEMG activation. This research demonstrated that ten healthy subjects were able to reduce the activation of their soleus muscle by ~35% when using the exoskeleton. It is important to note that, despite recent advancements in developing lower-body exoskeletons aimed at reducing the metabolic cost of gait, these recent systems predominantly employ non-neural control methods (Nuckols et al., 2020; Kim et al., 2019; Lim et al., 2019), rather than neural approaches such as EEG or EMG. This is not necessarily the case for lower body exoskeletons developed for other applications.

Finally, work on NM-controlled supernumerary legs is relatively limited in the literature. Parietti and Asada (2017) proposed a pair of supernumerary legs worn at the hip, with a wide and hemispheric workspace and capable of fully supporting the weight of the user. These legs were controlled by two sEMG signals from the torso and two from the chest muscles. A contraction of the right-side muscles moved the right leg forward (chest muscle) or backward (torso muscle), and similarly for the left leg. Muscles on the upper body were used to control the extra limbs without interfering with the natural limbs, thus enabling independent control of the supernumerary legs. Subjects were able to achieve accurate and independent control of the supernumerary legs. A summary of lower body WA systems with their features is presented in Table 3.

**Table 3:** Specifications summary of lower body WAs.

**Device name**	**Control modality**	**Measurement site**	**Target population**	**Attachment**	**Year**
Robotic ankle exoskeleton (Gordon and Ferris, 2007)	sEMG	Soleus muscle	Stroke and spinal cord injury patients	Placed below the knee and to the foot	2007
Robotic ankle exoskeleton (Koller et al., 2015)	sEMG	Soleus muscle	Healthy subjects	Placed below the knee and to the foot	2015
Extra robotic limbs (Parietti and Asada, 2017)	sEMG	Pectoralis major, rectus abdominis	Healthy subjects	Placed at sides of legs attached with a harness on the waist and hips	2017
Leg enhancer (Cenit and Gandhi, 2020)	sEMG	Vastus lateralis, rectus femoris, tibialis anterior, soleus and others	Elderly	Placed on the whole leg	2020
Gait rehabilitation exoskeleton (Chen et al., 2023)	sEMG	Quadriceps femoris and hamstrings	Stroke and spinal cord injury patients	Exoskeletal frame placed on the waist, hip, and knee joints	2023
EEG-oneDoF (Noda et al., 2012)	EEG	64 channels (10–20 system)	Stroke and spinal cord injury patients	Placed on legs and feet with chest harness	2012
NeuroRex (Contreras-Vidal and Grossman, 2013)	EEG	64 channels (10–20 system)	Mobility-impaired people	Legs and the waist	2013
Lower body exoskeleton (Kilicarslan et al., 2013)	EEG	64 channels (10–20 system)	Individuals with tetraplegia or paraplegia	Placed on legs and feet with chest harness	2013
Robotic gait orthosis (Do et al., 2013)	EEG	64 channels (10–20 system)	Individuals with tetraplegia or paraplegia due to spinal cord injury	Placed on legs and feet with chest harness	2013
Robotic-assisted treadmill walking (Garćıa-Cossio et al., 2015)	EEG	64 channels (10–20 system)	Stroke patients	Placed on legs and feet with chest harness	2015
Lower limb exoskeleton control system (Kwak et al., 2015)	EEG	Occipital cortex	Patients with motor disabilities	Placed on legs and feet with waist harness	2015
Brain-machine interface-based gait protocol (Donati et al., 2016)	EEG	Contralateral motor cortex	Spinal cord injury patients	Placed on legs and feet with chest harness	2016
Ambulatory exoskeleton (López-Larraz et al., 2016)	EEG	32 channels (10-20 system)	Individuals with incomplete paraplegia	Attached on both legs	2016
Brain-controlled exoskeleton (Lee et al., 2017)	EEG	Contralateral motor cortex	Individuals with tetraplegia	Attached on both legs	2017
BMI lowe limb exoskeleton (Ferrero et al., 2021)	EEG	27 electrodes	Patients with motor disorders	Attached on both legs	2021
Gait Rehabilitation HRI (Gui et al., 2017)	sEMG; EEG	Rectus femoris, semitendinosus muscles; Occipital cortex	Individuals with paraplegia	Placed on the whole leg	2017
mHMI lower limb exoskeleton (Gordleeva et al., 2020)	sEMG; EEG	Fasciae latae, rectus femoris, and others; Motor cortex	Patients with motor disorders	Attached on both legs	2020

### 3.4 Commercial products

There are several commercially available WAs designed for rehabilitation and enhancement. For example, the Lokomat is a robotic device that assists patients with walking difficulties by providing targeted gait training using virtual reality, facilitating recovery through an adjustable exoskeleton that supports the legs during therapy sessions (Hocoma, 2023). Another example is REX, a hands-free robotic WA that enables users to stand, walk, and engage in therapeutic exercises without crutches (Rex Bionics, 2023). However, both Lokomat and REX, like many similar products, rely on control paradigms that are not NM-based.

NM-controlled WAs are a promising but still emerging technology. While sEMG and EEG can offer reliable control, this is often limited to controlled research environments. Many NM-controlled systems in the literature integrate the hardware of commercially available products with novel NM-control paradigms to develop more advanced solutions.

There are a few commercial WAs that do utilize sEMG for control. One example is the HAL Lower Limb, a medical device designed to assist individuals with neuromuscular diseases, spinal cord injuries, and stroke. By detecting sEMG signals from the skin's surface, it translates the wearer's neural intentions into movement, enhancing mobility and rehabilitation (Lower Limb HAL, 2023). Similarly, the HAL Single Joint Type is a compact, lightweight robotic device focused on joint-specific rehabilitation, targeting areas like the elbows, knees, shoulders, and ankles, also controlled via sEMG (Single Joint HAL, 2023).

While there are few NM-controlled WAs utilizing sEMG, there are no commercially available WAs that rely primarily on EEG for control. EEG-based control remains largely within research settings, as the technology is still in development for real-world applications. This highlights a significant gap in the commercialization of EEG-based WAs, which could potentially offer more intuitive control for users.

### 3.5 Summary of findings

The summary of findings on NM-controlled WAs highlights several key points. Firstly, NM-controlled upper body and lower body exoskeletons are among the earliest implementations of WAs, with a substantial body of literature documenting these developments. In terms of supernumerary limbs, supernumerary fingers stand out as both the earliest and most researched, given that hands are the most utilized body part for physical interaction with the world. Conversely, supernumerary legs and arms have received less attention, likely due to the more complex design challenges they present.

Furthermore, it is observed that EEG is frequently used for lower body exoskeleton control, while sEMG is commonly used for upper body and hand WAs. This could be due to how EEG activations linked to larger natural effectors, such as legs, are more easily distinguished from effectors that might require finer control, such as hands and fingers. In addition, sEMG could be the more useful counterpart for the latter two types of NM-controlled WAs when considering that upper body and hands are often associated with tasks that require finer adjustments in muscular torque. Nonetheless, there is a rising trend of using both EEG and sEMG as a hybrid control modality. These implementations use EEG for intent detection and sEMG for force estimation, or switch between modalities upon detecting fatigue. Additionally, as EEG classification methods improve, distinguishing neural activity associated with smaller effectors, such as fingers, is becoming possible. This advancement suggests the potential for hybrid EEG-sEMG systems to achieve finer control in NM-controlled WAs.

**Figure 5:**
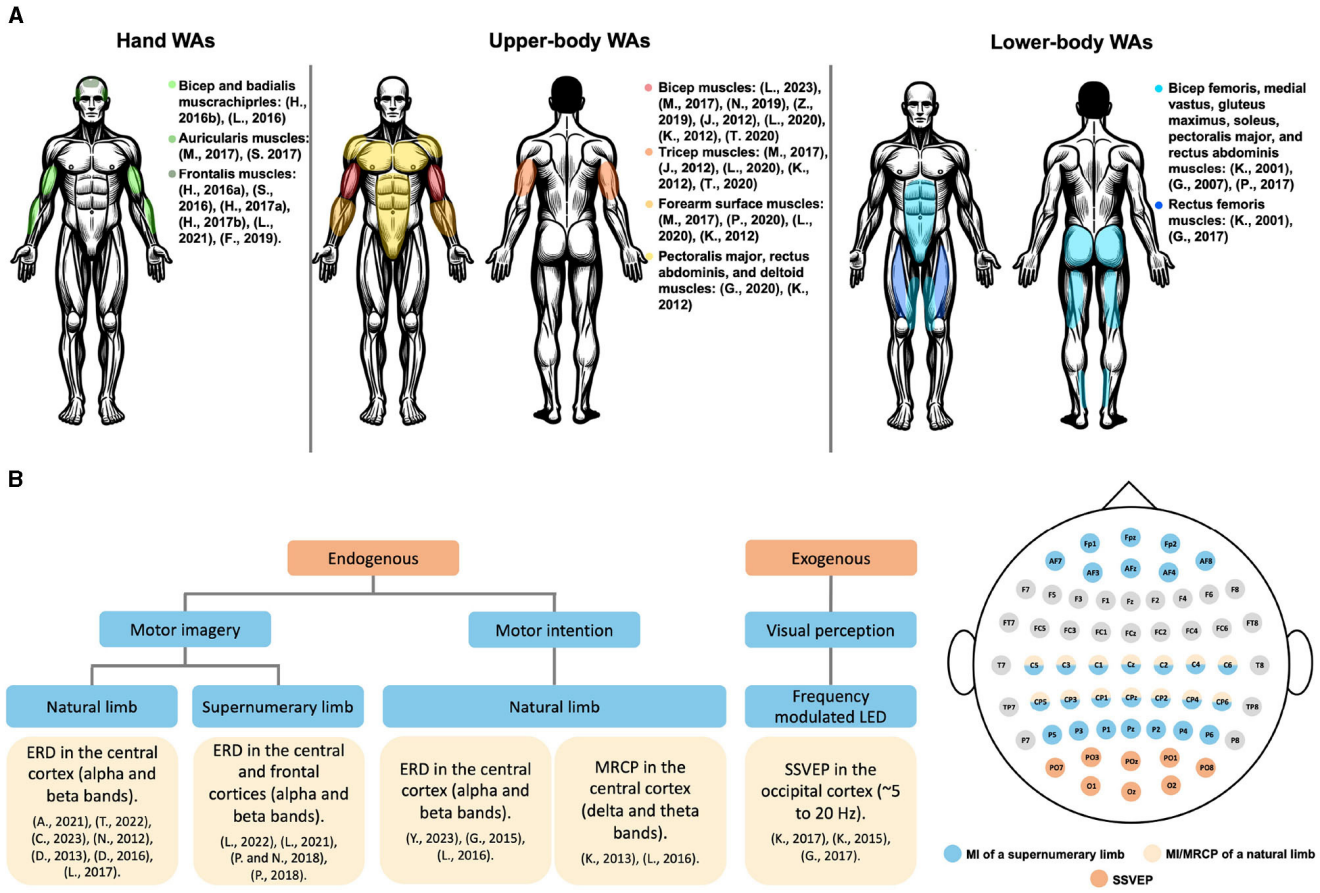
Pictorial summary. **(A)** An illustration depicting the muscles utilized for acquiring sEMG signals, depending on the type of WA, which are frequently employed in WA control. **(B)** Employed EEG signals for WAs control and their acquisition locations on the scalp. MI, motor imagery; MRCP, movement related cortical potentials; SSVEP, steady-state visually evoked potentials.

Additionally, a summary of the commonly used muscles for sEMG signal generation toward controlling each of the three types of WAs is shown in Figure 5A. Depending on the application and other design considerations, the choice of the muscle varies. It can be observed that muscles used to control hand WAs are located on the arms, forehead, or around the ears. For upper body WAs, muscles located at the chest, abdomen, and arms are commonly employed. On the other hand, lower body WAs commonly utilize muscles of the abdomen or legs for sEMG-based control. As for EEG based control, a summary of the commonly used cortical activations toward controlling WAs based on the EEG signal type is shown in Figure 5B. These signals are not specific to the type of WA employed; instead, they are related to the eliciting neural mechanism. MI of WAs elicits ERDs in the central and frontal areas. MI and movement-related cortical potentials (MRCPs) of natural limbs, used to control WAs, are commonly detected in the central cortex. Lastly, SSVEP in the occipital cortex, elicited in response to visual flickering stimuli, has proven beneficial in controlling WAs.

It is interesting, but reasonable, to note that MRCPs are largely linked to those of natural limbs, rather than supernumerary ones. The intent to move differs from MI, arguably more intuitive than its counterpart. Thus, it is more likely to be observed for natural effectors, which already possess cortical representations that can be easily accessed. On the other hand, supernumerary effectors are by nature foreign to the user's natural body representation, thus requiring a high degree of embodiment even for MI. An extended duration of training, as well as considerations for embodiment, would likely be required in order to move from MI to motor intention for supernumerary limbs, if indeed this shift is feasible.

This leads to another key observation, that sensory feedback is often an underemphasized element in NM-controlled WA design. For instance, only one upper body WA was found to employ sensory feedback in its design (Cisnal et al., 2023). On the other hand, few examples of hand WAs were found to employ sensory feedback to convey proprioceptive as well as tactile information. A summary of NM-controlled WAs incorporating sensory feedback is summarized in Table 4. Finally, minimal research exists on evaluating the effects of prolonged use of WAs, particularly examining the long-term neuroplasticity and embodiment effects of these devices.

**Table 4:** Summary of literature on WAs that employ sensory feedback in their design.

**Device name**	**Augmentation type and control modaliity**	**Measured parameter**	**Feedback modality**	**Feedback assessment**
Robotic extra thumb (RET) (Meraz et al., 2017)	*Hand WA, sEMG*	Contact force Finger position	Electrical stimulation Visual stimulation	Reduced error rate and the increased number of successful contacts between the RET and the other fingers.
sEMG-based robotic thumb (Aoyama et al., 2019)	*Hand WA, sEMG*	Finger position	Vibrotactile stimulation through phantom sensation	Reduced error rate and the increased number of successful contacts between the Robotic Thumb and the other fingers
Extra robotic thumb (ERT) (Shikida et al., 2017)	*Hand WA, sEMG*	Joint angle	Vibration stimulation	Increased number of successful contacts between the ERT and the other fingers
sEMG-based robotic sixth finger (Franco et al., 2019)	*Hand WA, sEMG*	Movement intent	Vibration stimulation	Reduced completion time, reduced muscle effort, and self report.
EEG-oneDoF (Noda et al., 2012)	*Lower body WA, EEG*	Intended movement	Visual stimulation	NA
Brain-machine interface-based gait protocol (Donati et al., 2016)	*Hand WA, sEMG*	Contact force	Tactile stimulation	Improved somatic sensation
	*Hand WA, sEMG*	Intended movement	Visual stimulation	
Brain-controlled exoskeleton (Lee et al., 2017)	*Hand WA, sEMG*	Intended movement	Visual stimulation	Reduced completion time
RobHand exoskeleton (Cisnal et al., 2023)	*Hand WA, sEMG*	Movement intent	Visual stimulation	Improved accuracy in performing required gestures

## 4 Outlook

### 4.1 Challenges and future work

The field of NM-controlled WAs, situated at the intersection of various disciplines, introduces multiple dimensions of challenges. Each challenge presents an opportunity for a future work. Below, we list some of these persistent challenges and possible directions for overcoming them:

- Consistency of performance: Research has shown that physiological signals, including EEG and sEMG, contain personalized elements, leading to variations in neural correlates from one individual to another. A significant challenge in NM-controlled WAs is the consistency of performance across different users. Developing calibration and fine-tuning algorithms to personalize these NM-controlled WAs represents a promising direction for future research, potentially utilizing big neurophysiological data to train AI based solutions.
- Context-aware control: WA technologies must be equipped with capabilities to perceive and interpret contextual information from the environment, such as user intentions, task requirements, spatial awareness, and social cues. By leveraging this context, WAs can dynamically adjust their behavior, control strategies, and interaction modalities to better assist, collaborate, and communicate with the user. A key challenge is the development of robust context recognition and understanding algorithms that can accurately interpret complex user and environmental cues. Another challenge is the design of efficient control strategies that can seamlessly integrate the context-aware information with the user's natural movements and intentions. One possible direction is the integration of multimodal sensing technologies (augmenting EEG-sEMG sensing with visual, auditory, and tactile sensors) to capture context-rich information and utilize machine learning algorithms to offer shared control of the WA. Furthermore, advances in machine learning offer exciting solutions for WA control. A recent example presents a deep learning method for predicting finger forces from motor units in the forearm using unsupervised approach. This eliminates the need for labeled finger force data during training, making it applicable in cases where force measurements are unavailable, such as in individuals with amputations (Meng and Hu, 2024).
- User-centered and ergonomic design: The need for user-centered and ergonomic WA is widely recognized. The number of WA in daily use is still very low not only due to missing availability on the market but also due to usability challenges. Personalized WA technologies that can be used by specified users to achieve specified goals with effectiveness, efficiency, and satisfaction in a specified context of use are far from being a reality. Involving all stakeholders, including the end user, throughout the development process to address human/environmental factors is essential. Physical and psycho-social factors, such as physical abilities, varying skills, knowledge, prior experiences or expectations must be investigated and valued as design criteria. In addition, ergonomic factors, such as muscular fatigue, stress on the musculoskeletal system, and technology acceptance should also be considered through self-reports, observational, and cognitive methods. Performing ergonomic risk assessments in order to evaluate the impact of WA technologies on the health and wellbeing, such as the development of musculoskeletal disorders is essential. Long term ergonomic effects should also be examined and incorporated in the design process.
- Ethical considerations: WAs raise several ethical concerns that need to be navigated by several entities in the community such as medical professionals, WAs designers, stakeholders, legislators, and others. Potential ethical challenges and concerns include safety, security, data privacy, accessibility and equity, and long-term neural effects such as manipulation of body representation in the brain. Safety considerations include safeguarding against hazards from power sources such as batteries, preventing sudden and un-intentional high force exertions, ensuring protection against falls in lower body exoskeletons, and avoiding collisions with obstacles. There is a pressing need for personal care robots' standards (such as ISO 13482) to be tailored for WAs. As for security, it is vital to protect these devices from unauthorized access and control by entities other than the intended user, developing layers of security. Unequal access to WA technologies, due to high cost of the technology, raises concerns about fairness and equity. Such disparities could significantly affect individuals' opportunities to compete in the society. Excluding marginalized communities can be seen as morally problematic and has to be addressed by developers and legislators (Oertelt et al., 2017). Finally, there are several neural complications that might arise due to and during the usage of WAs, many of those are discussed in the literature (Dominijanni et al., 2021). One possible complication is altering the body representation in the brain and thus impairing individuals from using their natural limbs to their full-extent.

### 4.2 Trends

Finally, being in its early stages, the field of NM-controlled WAs is ripe with various trends presenting exciting opportunities. Below, we present some of these emerging trends:

- WA evaluation: developing WA evaluation methods, based on self-reports, performance, behavior, and cognitive factors, in order to evaluate the impact of WA technologies on the health and wellbeing is essential. Furthermore, the assessment of the long-term use of WAs and its impact on the neural body map in realistic and practical environment represents a venue for exploration.
- Multimodal fusion: given how WA operate in unstructured environments and interact closely with humans, incorporating multi-modal sensors enables WAs to accumulate and process information from diverse sources, leading to enhanced reliability and usability. Furthermore, multimodal sensory feedback (such as proprioceptive and cutaneous) offers the potential to improve embodiment and influence plasticity in order to improve performance. Developing algorithms for information fusion warrants the enhancement of motor performance and user satisfaction.
- Soft WA: designing soft WAs represents a promising direction toward creating more user-friendly and ergonomic devices. Recent advancements in soft robotics and material science could lay the groundwork for this development.
- MI training paradigms: in order to improve the performance of neuromotor WA control, MI training paradigms must be developed to reliably and effectively induce distinct MI signatures for the augmentation. Such a training paradigm would involve a combination of motor observation, motor execution, and MI exercises using virtual and physical environments. Furthermore, training paradigms should explore the use of positive sensory feedback to promote kinesthetic MI functions to improve the clarity of MI and the overall motor performance, as well as the embodiment of the WA device. Finally, the neurological changes in MI and body representation over time should be investigated. Machine and deep learning models can be utilized to extract MI signatures from EEG data and adapt the positive sensory feedback in order to accelerate the MI training.
- AI augmentation: with a promise to enable machines to perform tasks that require human intelligence, AI has become a hot research topic in recent years. Based on the results of this review, most of the contemporary WA technologies are not equipped with the computing power for AI augmentation in order to fuse multimodal data from various sensing systems. Advanced AI algorithms are needed to fuse the collected multimodal raw data and understand the user and environmental context. Furthermore, AI methods can monitor the health of the WA device. Repair and maintenance can then be triggered automatically upon detecting a malfunction. Finally, with the success of using large language models in many applications, integrating linguistic capabilities to the WA could revolutionize the way we interact with WAs (Yu et al., 2024).
